# Development of a Semiglobal Reaction Mechanism for the Thermal Decomposition of a Polymer Containing Reactive Flame Retardants: Application to Glass-Fiber-Reinforced Polybutylene Terephthalate Blended with Aluminum Diethyl Phosphinate and Melamine Polyphosphate

**DOI:** 10.3390/polym10101137

**Published:** 2018-10-12

**Authors:** Yan Ding, Stanislav I. Stoliarov, Roland H. Kraemer

**Affiliations:** 1 Department of Fire Protection Engineering, University of Maryland, 3106 J.M. Patterson Building, College Park, MD 20742, USA; yanding@umd.edu; 2 Flame Retardancy & Performance Polymers Research, BASF Advanced Chemicals Co., Ltd., No 300 Jiangxinsha Road, Pudong District, Shanghai 200137, China; roland.kraemer@basf.com

**Keywords:** polymer combustion, pyrolysis, flame retardants, thermal analysis, inverse modeling, ThermaKin

## Abstract

This work details a methodology for parameterization of the kinetics and thermodynamics of the thermal decomposition of polymers blended with reactive additives. This methodology employs Thermogravimetric Analysis, Differential Scanning Calorimetry, Microscale Combustion Calorimetry, and inverse numerical modeling of these experiments. Blends of glass-fiber-reinforced polybutylene terephthalate (PBT) with aluminum diethyl phosphinate and melamine polyphosphate were used to demonstrate this methodology. These additives represent a potent solution for imparting flame retardancy to PBT. The resulting lumped-species reaction model consisted of a set of first- and second-order (two-component) reactions that defined the rate of gaseous pyrolyzate production. The heats of reaction, heat capacities of the condensed-phase reactants and products, and heats of combustion of the gaseous products were also determined. The model was shown to reproduce all aforementioned experiments with a high degree of detail. The model also captured changes in the material behavior with changes in the additive concentrations. Second-order reactions between the material constituents were found to be necessary to reproduce these changes successfully. The development of such models is an essential milestone toward the intelligent design of flame retardant materials and solid fuels.

## 1. Introduction

The combustion of polymeric materials can be characterized as a coupling between gas-phase and condensed-phase phenomena. The condensed-phase phenomena, broadly referred to as pyrolysis, have been less understood due to their inherent complexity and a lack of experimental capabilities for direct monitoring of relevant chemical transformations [1,2]. The kinetics of thermal decomposition is, perhaps, the most important characteristic of the pyrolysis process [3]. This kinetics is usually derived from a global measurement, such as thermogravimetric analysis (TGA), which provides information on mass loss from a thermally thin sample exposed to a linear temperature ramp.

Isoconversional methods [4,5,6], which require TGA data collected at multiple heating rates and/or temperatures, have been widely used to determine kinetic parameters of decomposition. These methods utilize a single (global) reaction to represent the overall decomposition process. The complexity of decomposition, which may include thousands of elementary steps, is captured via a variation of activation energy with the extent of conversion. One significant drawback of these methods is that they do not naturally extend to multicomponent materials, especially when components chemically interact with each other at elevated temperature.

In addition to global kinetic models, several highly detailed mechanisms of thermal decomposition have been developed for a few commodity plastics [7,8]. These mechanistic models include hundreds of species and thousands of elementary reactions and provide a molecular-level insight into the decomposition process. These models have also been demonstrated to capture cross-polymer chemical interactions in binary blends undergoing pyrolysis [9,10,11]. The main drawback of such models is the formidable effort required for their construction and validation. They also require estimation of a large number of kinetic parameters which are impossible to measure directly. Finally, the high computational cost of these models precludes their use in simulations of large physical systems.

Over the past several years, our group has been developing an approach where polymer decomposition is modeled using a small number (1 to 15) of Arrhenius reactions of the first or second order [12,13,14,15,16,17,18]. This feature of our approach is not unique. A number of researchers constructed their solid fuels decomposition models from a small number of consecutive or parallel reactions [1,19,20]. The resulting model is frequently referred to as a semiglobal or lumped-species model. The distinguishing feature of our approach is that it is not based on TGA data alone but also relies on fully quantitative Differential Scanning Calorimetry (DSC) and Microscale Combustion Calorimetry (MCC) measurements. Using an iterative process, the simplest reaction model that simultaneously captures the results of all of these measurements is constructed and multiple model parameters including stoichiometric coefficients, activation energies, pre-exponential factors, heat capacities of the condensed-phase reactants and products, heats of melting and decomposition, and heats of combustion of the gaseous pyrolyzate are derived.

**Figure 1:**
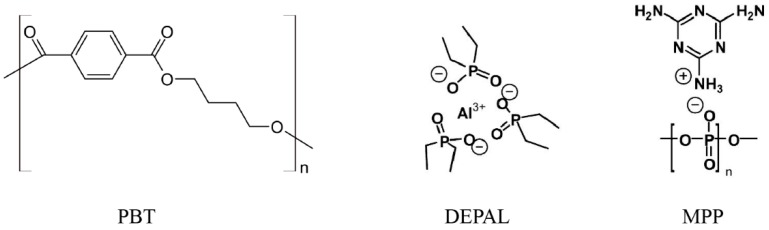
Chemical structures of polymer (polybutylene terephthalate (PBT)) and two flame retardant additives (aluminum diethyl phosphinate (DEPAL) and melamine polyphosphate (MPP)).

In a recent publication [18], we showed evidence indicating that it is possible to extend this methodology to a binary mixture of reactive components and create a semiglobal reaction mechanism that successfully captures the degradation behavior over a broad range of mixture compositions. In the current study, we provide further validation of this assertion by demonstrating that this approach can be extended to tertiary reactive blends. The subject of the current investigation is polybutylene terephthalate (PBT) reinforced with glass fiber (GF) and blended with aluminum diethyl phosphinate (DEPAL) and melamine polyphosphate (MPP). The chemical structures of the polymer and the two flame retardant additives are shown in Figure 1.

PBT is widely used in electrical systems, including lamp holders, switches, circuit breakers, and motor casings. It has high heat resistance, mechanical strength, and water resistance, and excellent electrical insulating properties [21]. However, pure PBT is flammable. GF is added to PBT to prevent dripping and to help maintain integrity upon burning. Phosphorus-based compounds are added to PBT to improve fire resistance [22,23,24]. Melamine derivatives (such as MPP) have been shown to act as a synergist to phosphorus flame retardants to further enhance the fire resistance of the blends [24,25,26].

Several studies have been conducted to understand the pyrolysis and fire behavior of flame retardant PBT/GF [22,24]. Braun et al. [22] found that an addition of 13–20 wt % of DEPAL in combination with melamine cyanurate improves the fire retardancy of PBT/GF significantly (UL-94 rating of V0 and limiting oxygen index above 42% were achieved). DEPAL was hypothesized to act primarily through a release of phosphinate compounds that inhibited the chemistry of the gas-phase combustion. It was also noted that DEPAL increases carbonaceous residue (char) production and thus creates an additional thermal barrier and further improves mechanical stability of the material exposed to flame. None of the studies provided a quantitative relation between the thermal decomposition behavior of PBT/GF blends and flame retardant content. Knowledge of this relation is critical for the design of materials with the optimum combination of flame resistance and mechanical properties tailored for specific applications [27].

## 2. Materials and Methods

### 2.1. Materials

The materials studied in this work were provided by BASF (Ludwigshafen am Rhein, Rhineland-Palatinate, Germany). Compounds were produced in a twin-screw extruder and subsequently injection-molded [28] into 3.8 mm thick plates. Their compositions are shown in Table 1. The top five blends were used to build the reaction mechanism; the last three were employed to demonstrate the mechanism’s ability to extrapolate beyond the compositions used in the building process. Additional experiments were conducted on pure MPP (not listed in Table 1) supplied in the form of powder. The results of these experiments were also used in the mechanism construction.

The samples for TGA, DSC, and MCC tests were prepared by either cutting or grinding the plates into 3–7 mg specimens. Each test was repeated multiple times using varying sample mass and shape to verify that the test results were not sensitive to these factors and thus ensure thermally thin behavior (i.e., no significant temperature or composition gradients within the sample). All samples were conditioned in a desiccator in the presence of Drierite for a minimum of 48 h prior to testing to obtain measurements with negligible contribution from moisture.

### 2.2. Simultaneous Thermal Analysis (STA)

449 F3 Jupiter Simultaneous Thermal Analyzer (Netzsch Geratebau GmbH, Selb, Bavaria, Germany) was employed to conduct TGA and DSC tests simultaneously. All STA tests followed a carefully prescribed temperature program. The temperature program for these tests had a conditioning period in which the sample was maintained at 313 K for 25 min, followed by a linear heating up to 873 K. The majority of the tests were conducted at a nominal heating rate of 10 K·min^−1^ to ensure that the heating rate was sufficiently low to decouple the chemical reactions from the mass and thermal transport. The furnace was continuously purged with nitrogen at a flow rate of 50 mL·min^−1^ to guarantee an anaerobic environment. Platinum–Rhodium crucibles with lids containing a small opening were utilized to maximize heat flow sensitivity and temperature uniformity. The sample mass and heat flow data were collected as a function of time and pyrolyzing sample temperature. An empty crucible baseline was collected prior to every test and subtracted from the corresponding data obtained with a sample. A detailed description of the instrument calibration and testing protocol can be found in an earlier publication [12].

The STA tests of the five blends used in the reaction model development (see Table 1) were conducted at 10 K·min^−1^ and were repeated ten times to ensure reproducibility and accumulate the necessary statistics. Additional tests of these blends were performed at 5 and 20 K·min^−1^. These tests were repeated three times and were used to verify that the developed reaction model correctly extrapolates the material’s behavior to alternate thermal conditions. Only TGA data were collected in these additional tests because of their significantly higher reproducibility. The STA tests on the remaining three (model validation) blends were conducted at 10 K·min^−1^ and repeated five times. Finally, the TGA tests of MPP were performed at 10 K·min^−1^ in triplicate.

### 2.3. Microscale Combustion Calorimetry

MCC is a standardized test method used to measure the heat release rate (HRR) from complete combustion of gaseous products evolved from a pyrolyzing solid. The MCC tests were performed at the same prescribed heating rate of 10 K·min^−1^ as used in the STA tests to enable a direct comparison with the STA measurements. A slightly higher temperature of 348 K was set as the initial temperature. All the samples were pyrolyzed in a ceramic crucible without a lid. The gaseous pyrolyzate was purged by nitrogen at a flow rate of 80 mL·min^−1^ into the combustor, where the pyrolyzate was mixed with excess oxygen, supplied at a flow rate of 20 mL·min^−1^, and oxidized. The temperature of the combustor was set at 1173 K to ensure completeness of the combustion process. The heat released from the combustion process was measured using the principle of oxygen consumption [29]. The HRR was measured as a function of time and pyrolyzing sample temperature. The MCC apparatus was carefully calibrated following the recommended procedures [29]. The MCC tests were performed on the materials used in the reaction model development. Each test was repeated three times to ensure reproducibility.

### 2.4. Modeling

ThermaKin [30,31] is a numerical modeling environment that was developed to compute the transient rate of gaseous fuel production by a pyrolyzing solid. Non-steady mass and energy conservation equations are solved by ThermaKin to account for the fundamental physical and chemical processes occurring in the condensed phase during pyrolysis. First- and second-order (two-component) chemical reactions are represented in ThermaKin using the Arrhenius expression for the reaction rate constants. ThermaKin was employed in this study to inversely model the TGA, DSC, and MCC data to determine the kinetics and thermodynamics of the thermal decomposition as well as the heats of combustion of the gaseous decomposition products.

In the current model setup, all samples were treated as thermally thin and simulated using a single element (i.e., no temperature or composition gradients inside the samples). The convection coefficient at the element boundary was defined as sufficiently high (1 × 10^5^ W·m^−2^·K^−1^) to ensure that the element’s temperature follows the corresponding mean experimental temperature profile. This profile was approximated by expressing the heating rate as an exponentially decaying sinusoidal function. This function was used to correctly account for deviations of the instantaneous experimental heating rate from the nominal (or set) rate with time, as explained in detail in earlier publications [15,17]. The mass flow boundary condition was defined such that all gaseous decomposition products escaped the element instantaneously.

## 3. Results and Discussion

### 3.1. Overall Approach to Reaction Model Development

The development of a generalized reaction model for the PBT/GF/DEPAL/MPP blends consisted of five steps. In the first step, a PBT decomposition model was derived from the TGA and DSC data obtained for the PBT/GF25 blend. The GF component was assumed to be nonvolatile and chemically inert.

In the second step, an attempt was made to simulate the TGA and DSC data obtained for the PBT/GF/DEPAL blends using a combination of the PBT decomposition model and DEPAL decomposition model, which was based on information available in the literature [32]. Significant differences between these simulations and experimental data were identified. These differences were interpreted as evidence of a chemical interaction between the polymer matrix and DEPAL. The reaction parameters of this interaction were initially estimated through a fitting of the TGA and DSC data obtained for PBT/GF25-DEPAL8 (a blend with a low concentration of DEPAL) and further adjusted by fitting of the data collected for PBT/GF25-DEPAL16 (a blend with a high concentration of DEPAL) until the best compromise in representing both datasets was achieved. In the third step, the same procedure was used to develop a reaction mechanism for the PBT/GF/MPP blends.

In the fourth step, the combined reaction model (including PBT, DEPAL, and MPP decomposition, as well as PBT–DEPAL and PBT–MPP interactions) was employed to simulate experimental MCC data for all blends used for the reaction model development (see Table 1). Heats of combustion of all gaseous products were derived from these simulations. In the final step, the combined reaction model was validated by testing its ability to predict TGA and DSC data at heating rates and for material compositions not used in the model development process.

### 3.2. Inverse Modeling of TGA and DSC Data for PBT/GF25

**Figure 2:**
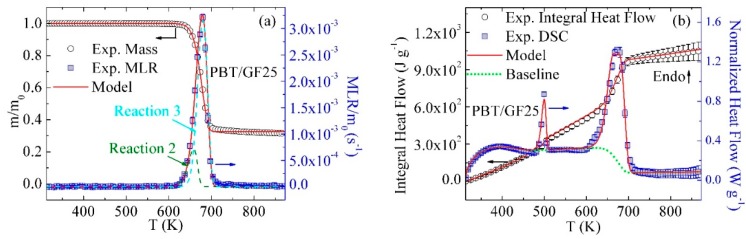
Experimental and simulated (**a**) TGA and (**b**) DSC data obtained for PBT/GF25 at 10 K·min^−1^.

The mean experimental results of TGA and DSC tests conducted on the PBT/GF25 material are shown as symbols in Figure 2. Figure 2a shows the dependence of sample mass, m, and mass loss rate, MLR, normalized by the initial mass, m_0_, on temperature, T. Figure 2b contains the initial-sample-mass normalized heat flow and integral heat flow as a function of temperature. The error bars were calculated from the scatter of the experimental data as two standard deviations of the mean (some error bars are comparable in size to the data symbols and, therefore, not shown). The experimental MLR profile contains a single peak at 680 K corresponding to the thermal decomposition process. The decomposition produced 31.5 wt % of residue including 25 wt % of GF. The heat flow curve contains two distinct maxima. The first (500 K) and second (680 K) maxima represent the melting and decomposition processes, respectively.

Inverse modeling of the TGA and DSC data was performed using the approach described in detail in earlier publications [15,16,17,18]. Only a brief description is provided here. A single first-order reaction was initially employed to model the TGA data. The mass-based stoichiometric coefficients of this reaction were initially set to capture the residue yield observed in the TGA experiments. The Arrhenius parameters of this reaction were initially estimated using analytical expressions [33] relating these parameters to the temperature and height of the targeted MLR peak. Subsequently, these parameters were refined through a manually iterative process using ThermaKin until the modeling results were found to be in agreement with the experimental data. The reaction parameter manipulation was based on the following observations: an increase in the activation energy (E) shifted the MLR curve to a higher temperature and reduced the height of the peak; an increase in the pre-exponential factor (A) shifted the MLR curve to a lower temperature and increased the height of the peak. The agreement was declared to be satisfactory when the differences between the average experimental and modeled final mass residues and the temperatures and magnitudes of the MLR maxima were found to be within 3%, 5 K, and 8%, respectively. If it was impossible to achieve such agreement, an additional reaction was added to the reaction scheme and its parameters were adjusted iteratively to achieve further improvement.

In the case of PBT/GF25, two consecutive reactions, Reaction 2 and 3, were required to accurately capture the initial rise, maximum, and final decay of the MLR. These reactions are defined in Table 2. The component names used in the reaction definitions are self-explanatory. The reaction contributions to MLR profiles are depicted in Figure 2; their parameters are summarized in Table 3.

The heat flow data were analyzed by first focusing on the regions not associated with melting or decomposition. The data corresponding to these regions were divided by the instantaneous heating rate and fitted with linear functions representing heat capacities, c, of the corresponding condensed-phase components. The heat flow data between 313 and 460 K and 510 and 590 K were used to determine the heat capacities of PBT and PBT_Melt, respectively. The heat capacity of GF was obtained from the manufacturer [34]. The heat capacity of PBT_Res2 was impossible to resolve due to its small yield. Therefore, its heat capacity was assumed to be equal to the average heat capacity of chars produced by several common polymers—1700 J·kg^−1^·K^−1^ [13]. The heat capacity of the intermediate condensed-phase product (PBT_Res1) was assumed to be equal to the average heat capacity of the PBT_Melt and PBT_Res2. All heat capacity values are listed in Table 4.

The sensible heat flow baseline was subsequently calculated as a product of the mass fractions of condensed-phase components (whose temporal evolution was computed by ThermaKin), corresponding heat capacities, and instantaneous heating rate. The baseline obtained for the PBT/GF25 is shown as a green dotted line in Figure 2b. Subtraction of this baseline from the normalized experimental heat flow and subsequent integration of the differences yielded the values of the heat of melting and heats of decomposition, h. Reaction 1 (see Table 2) was added to the mechanism to simulate the melting process. The kinetics of this reaction and heats of melting and decomposition were refined until the overall heat flow curve simulated using ThermaKin was in agreement with the experimental data. This agreement was defined by the simulated heat flow maxima within 10%, temperatures of the maxima within 8 K, and the final integral heat flow value within 5% of the corresponding experimental data. The results of this exercise are shown in Figure 2b. The heats of melting and decomposition reactions are listed in Table 3.

### 3.3. Inverse Modeling of TGA and DSC Data for PBT/GF/DEPAL

**Figure 3:**
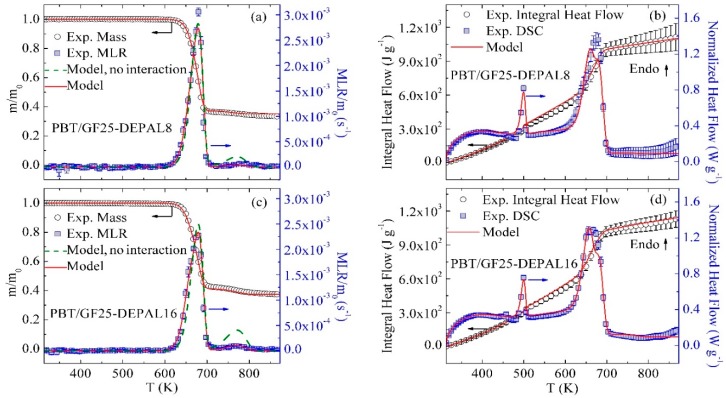
Experimental and simulated TGA and DSC data obtained for (**a**,**b**) PBT/GF25-DEPAL8 and (**c**,**d**) PBT/GF25-DEPAL16 at 10 K·min^−1^.

Figure 3 displays the mean experimental results of TGA and DSC tests on PBT/GF25-DEPAL8 and PBT/GF25-DEPAL16 material systems. With the introduction of DEPAL, the MLR profiles gain an additional, barely discernable peak at 765 K. This peak becomes somewhat more evident as the concentration of DEPAL increases to 16 wt %.

Duquesne et al. [32] investigated the decomposition of pure DEPAL using TGA which was conducted in nitrogen at a heating rate of 10 K·min^−1^. They found that DEPAL decomposed at about 750 K through a single step and produced 7 wt % of final residue. Given a similarity between the position of the second MLR peak observed in this study and that of pure DEPAL, this second peak was attributed to DEPAL decomposition. Reaction 4 (see Table 2) was added to the mechanism to represent this process. The stoichiometric coefficients of this reaction were determined from the results obtained by Duquesne et al. The kinetic parameters of Reaction 4 were estimated using the position of the second MLR peak (765 K) observed in the current experiments.

The results of the addition of Reaction 4 to the mechanism are shown as green dashed lines in Figure 3a,c. These simulations significantly overestimate the size of the second MLR peak for both blends, indicating that some DEPAL is consumed in a reaction with the polymer matrix prior to reaching its decomposition temperature. In addition, the experimental residue yields of the DEPAL-containing blends are notably higher than the values calculated based on the individual PBT and DEPAL contributions. The increased residue yields further support the existence of an interaction between the polymer matrix and DEPAL. The existence of this interaction was also noted in previous studies, which indicated that aluminum phosphinate terephthalate is formed due to a condensed-phase interaction between the polymer matrix and DEPAL [24,35].

To account for this interaction, a reaction between PBT_Melt and DEPAL—Reaction 5 (see Table 2)—was added to the mechanism. The stoichiometry and kinetics of this reaction were adjusted to correctly reproduce the experimental MLR peaks observed for the PBT/GF25-DEPAL8 and PBT/GF25-DEPAL16 blends. The final inverse modeling results using Reactions 1–5 are shown in Figure 3a,c as red solid lines; the reaction parameters are summarized in Table 3. The first simulated MLR peak is still primarily associated with the decomposition of PBT_Melt, only a relatively small fraction of which is consumed in the reaction with DEPAL. The second simulated peak is associated with the decomposition of the portion of DEPAL that did not react with PBT_Melt.

The experimental heat flow data obtained for PBT/GF25-DEPAL8 and PBT/GF25-DEPAL16 in the temperature range between 313 and 460 K were used to determine the heat capacity of DEPAL. The sensible heat contributions of PBT and GF were subtracted from the heat flow data to determine DEPAL’s contribution. The heat capacities of DEPAL_Res1 and PBT_DEPAL_Res1 components could not be determined from the heat flow data due to their small yields and were therefore assumed to be equal to the average heat capacity of chars produced by several common polymers—1700 J·kg^−1^·K^−1^ [13]. The heats of Reaction 4 and 5 were subsequently determined by fitting the heat flow data for both blends. These final heat flow modeling results are shown in Figure 3b,d. The values of heat capacities and heats of reaction are reported in Table 3 and Table 4, respectively.

### 3.4. Inverse Modeling of TGA and DSC Data for PBT/GF/MPP

**Figure 4:**
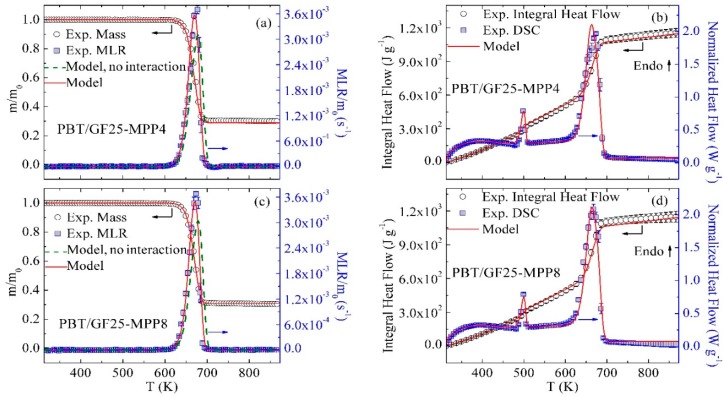
Experimental and simulated TGA and DSC data obtained for (**a**,**b**) PBT/GF25-MPP4 and (**c**,**d**) PBT/GF25-MPP8 at 10 K·min^−1^.

The mean experimental results of TGA and DSC tests of PBT/GF25-MPP4 and PBT/GF25-MPP8 blends are shown as symbols in Figure 4. With the incorporation of MPP into the GF-reinforced PBT, the main MLR peak increases in height (by 20% upon addition of 4 wt % of MPP) and becomes considerably more narrow in temperature. An attempt was made to capture this behavior by adding a reaction of decomposition of MPP, Reaction 6 (see Table 2), to the mechanism. This reaction was parameterized by matching the final residue yield and temperature of the main MLR peak observed in the TGA experiments conducted in this work on pure MPP. However, as indicated by green dashed lines in Figure 4a,c, the resulting model (no interaction) significantly underestimated the heights of the main MLR peaks; the initial rise and final decay of the MLR curves were not captured. Also, the final residue yields were underestimated by about 5%. It was further deduced that it is the reaction of decomposition of PBT_Res1 (Reaction 3) that was primarily responsible for the observed discrepancies. Therefore, a reaction between PBT_Res1 and MPP—Reaction 7 (see Table 2)—was added to the model to compete with Reaction 3. Finally, one more consecutive reaction, Reaction 8, was added to accurately capture the final decay of the MLR peaks observed for both blends. These inverse modeling results using Reactions 1–3 and 6–8 are shown in Figure 4a,c as red lines. The kinetic parameters of these additional reactions are summarized in Table 3.

The heat capacity of MPP was obtained in the same way as the heat capacity of DEPAL (see Section 3.3). The heat capacities of MPP_Res1 and PBT_MPP_Res2 could not be resolved and were assumed to be equal to the average heat capacity of chars produced by several common polymers—1700 J·kg^−1^·K^−1^ [13]. The heat capacity of PBT_MPP_Res1 was taken as the average heat capacity of PBT_Melt and PBT_MPP_Res2. The heats of Reaction 6, 7, and 8 were subsequently determined by fitting the heat flow data for both blends. The final heat flow modeling results are shown in Figure 4b,d. The values of heat capacities and heats of reaction are reported in Table 3 and Table 4, respectively.

### 3.5. Inverse Modeling of MCC Data

**Figure 5:**
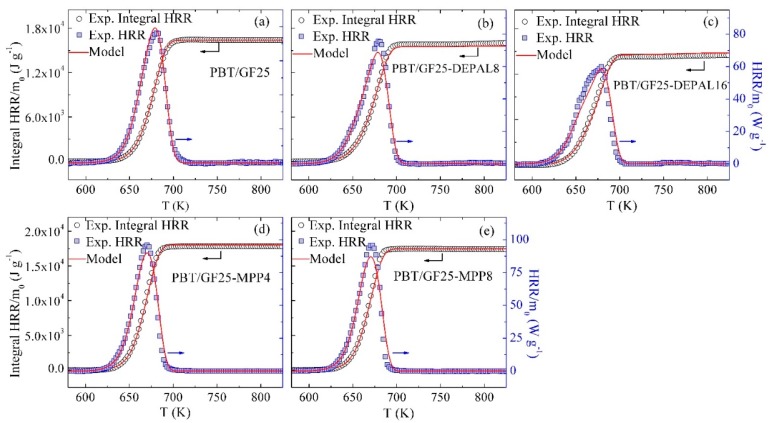
Experimental and simulated Microscale Combustion Calorimetry (MCC) data obtained for (**a**) PBT/GF25, (**b**) PBT/GF25-DEPAL8, (**c**) PBT/GF25-DEPAL16, (**d**) PBT/GF25-MPP4, and (**e**) PBT/GF25-MPP8 at 10 K·min^−1^.

The mean experimental HRR normalized by the initial sample mass and the integral HRR for PBT/GF25, PBT/GF25-DEPAL8, PBT/GF25-DEPAL16, PBT/GF25-MPP4, and PBT/GF25-MPP8 blends are shown as symbols in Figure 5. Initial comparisons between the experimental HRR and modeled MLR profiles generated using the heating rate temporal profiles specific to the MCC experiments revealed minor discrepancies that were attributed to sample/sensor temperature nonuniformities present in the MCC and associated with the use of open ceramic crucibles. To correct for these nonuniformities, the experimental HRR curves were shifted to a higher temperature by 5–10 K using the guiding principle that any detected heat release required a concurrent mass loss. The experimental data presented in Figure 5 are the shifted data; the original data are not shown because they are nearly indistinguishable from the shifted results.

The heats of combustion (h_c_) of all gaseous products were first set to a single value that yielded the final integral HRR equal to that observed in the experiments. Subsequently, individual h_c_ values were adjusted up or down to capture the shapes of the experimental HRR peaks. As in the case of inverse modeling of TGA and DSC experiments, the iterative process continued until the differences between the modeled and experimental data satisfied specific criteria. These criteria were defined as differences of less than 8% between the values of HRR maxima, less than 10 K between the temperatures of the maxima, and less than 8% between the final integral HRR values. The resulting heats of combustion that satisfy these criteria are given in Table 5. All h_c_ values are within the range $0.7\times 10^{7}–3.8\times 10^{7}$ J·kg^−1^, consistent with the hydrocarbon nature of these gases [36]. The final simulated MCC results are shown in Figure 5.

### 3.6. Model Performance at Different Heating Rates

**Figure 6:**
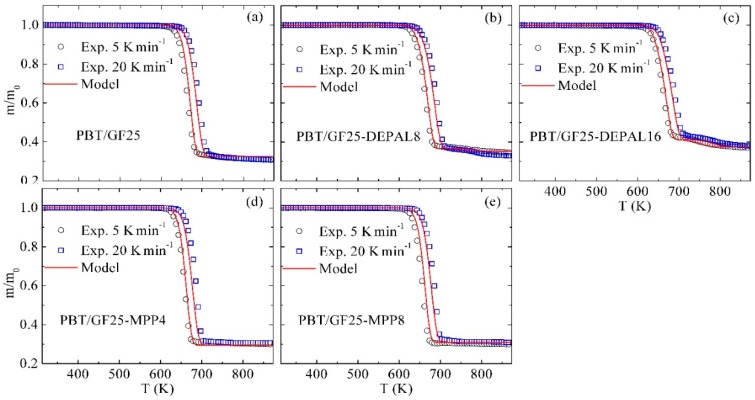
Experimental and simulated TGA data obtained for (**a**) PBT/GF25, (b) PBT/GF25-DEPAL8, (**c**) PBT/GF25-DEPAL16, (**d**) PBT/GF25-MPP4, and (**e**) PBT/GF25-MPP8 blends at 5 K·min^−1^ and 20 K·min^−1^.

The reaction mechanism shown in Table 2 and corresponding parameters reported in Table 3, Table 4 and Table 5 reproduce TGA, DSC, and MCC data collected at a nominal heating rate of 10 K·min^−1^ for PBT/GF25, PBT/GF25-DEPAL8, PBT/GF25-DEPAL16, PBT/GF25-MPP4, and PBT/GF25-MPP8 blends with the accuracy defined by the criteria discussed in Section 3.2 and Section 3.5. To further validate this reaction model, the mean experimental TGA data obtained at lower (5 K·min^−1^) as well as higher (20 K·min^−1^) heating rates on the same blends were compared with the corresponding predictions. This comparison is summarized in Figure 6; no MLR data are shown on this figure to avoid congestion. Overall, good predictions were obtained for all blends and heating conditions. The model slightly (by 5–8 K) underestimates the temperatures of the onset of the experimental mass loss at 20 K·min^−1^ for all blends, perhaps due to some minor nonuniformities in the sample/sensor temperature arising at this heating rate (and not corrected for by the STA temperature sensor calibration, which was carried out at 10 K·min^−1^).

### 3.7. Modeling of Different Material Compositions

**Figure 7:**
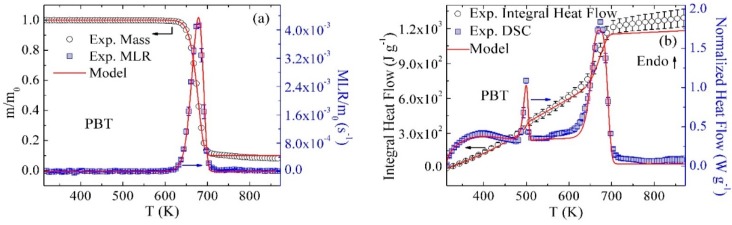
Experimental and simulated (**a**) TGA and (**b**) DSC data obtained for pure PBT at 10 K·min^−1^.

To validate the model assumption that GF acts as an inert additive, TGA and DSC experiments were performed on pure PBT samples. The results of these experiments are compared with the model predictions in Figure 7. The experimental TGA data are in nearly perfect agreement with the model. The experimental heat flow is slightly underpredicted toward the end of the experiment. This discrepancy is likely to be associated with the uncertainties in the high-temperature portion of the experimental heat flow baseline, which may not have been completely resolved in the five STA runs from which these data were derived (ten STA runs were used to generate all model calibration data).

**Figure 8:**
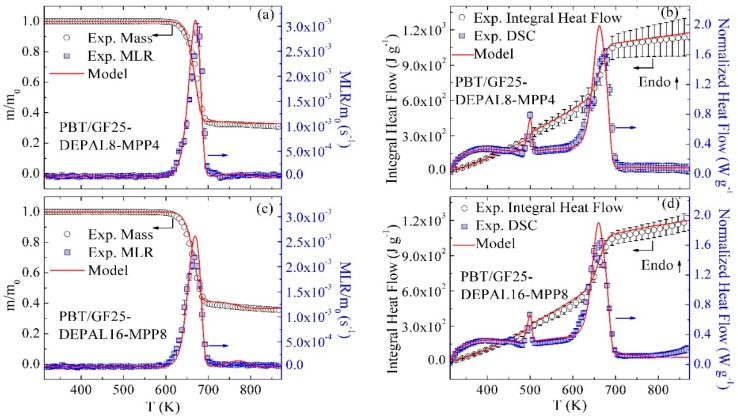
Experimental and simulated TGA and DSC data obtained for (**a**,**b**) PBT/GF25-DEPAL8-MPP4 and (**c**,**d**) PBT/GF25-DEPAL16-MPP8 at 10 K·min^−1^.

Finally, the predictions of the reaction model were compared with the experimental results obtained for material blends containing both flame retardants: PBT/GF25-DEPAL8-MPP4 and PBT/GF25-DEPAL16-MPP8. This comparison is shown in Figure 8. The model captures all TGA and DSC experimental data well, with the exception of a slight overprediction of the maximum heat flows. In addition to validating the model, this comparison indicates the absence of significant chemical interactions between DEPAL and MPP additives. The same conclusion was reached by Samyn and Bourbigot, who studied the thermal decomposition of DEPAL and MPP [37]. In the study by Samyn and Bourbigot, the thermal decompositions of DEPAL and MPP were characterized individually and the mass loss curve for a DEPAL–MPP blend was calculated based on the individual DEPAL and MPP contributions. The calculated mass loss curve agreed with the experimental data on the blend, which indicated the absence of chemical interactions between these additives in the condensed phase.

It should be noted that both the current work and the study by Samyn and Bourbigot did not account for the potential impact of DEPAL and/or MPP on the transport processes in the condensed phase. Analysis of this impact will be a subject of future work. Both DEPAL and MPP also affect the kinetics of the gas-phase combustion, which may explain a synergy between these additives observed in cone calorimetry tests [38].

## 4. Conclusions

A methodology for development of a quantitative reaction mechanism for a blend of reactive solids has been presented and demonstrated using a material system containing a GF-reinforced PBT compounded with flame retardants DEPAL and MPP. This methodology is based on TGA, DSC, and MCC experiments that are analyzed using inverse numerical modeling. The developed model was shown to simultaneously reproduce all measurement results for mixtures containing a full range of relevant flame retardant concentrations with a high degree of accuracy. While this model was based on a lumped species approach (and thus did not explicitly resolve individual chemical species), it did capture all essential aspects of the thermal decomposition behavior relevant to the flammability of studied materials. Moreover, through the presented analysis, key binary interactions between the material constituents were identified and implemented in the model using second-order (two-component) reactions.

A combination of the current reaction mechanism development methodology with a bench-scale heat and mass transport analysis described in detail elsewhere [15,16,39,40,41] will be required to develop comprehensive pyrolysis models for such reactive systems. This expansion of the current approach will be a subject of future work. Provided that such pyrolysis models can be successfully developed, they will provide a quantitative relation between material composition and its reaction to thermal insult. Development of such relations will enable optimization of passive fire protection of a built environment as well as maximization of useful energy output from composite solid fuels.

## Figures and Tables

**Table 1:** Composition of tested materials.

	Formulation Name	PBT (wt %) Ultradur B4500	GF (wt %) Glassfiber PPG 3786	DEPAL (wt %) Exolit OP 1240	MPP (wt %) Melapur 200
Model Development	PBT/GF25	75	25	0	0
	PBT/GF25-DEPAL8	67	25	8	0
	PBT/GF25-DEPAL16	59	25	16	0
	PBT/GF25-MPP4	71	25	0	4
	PBT/GF25-MPP8	67	25	0	8
Model Validation	PBT	100	0	0	0
	PBT/GF25-DEPAL8-MPP4	63	25	8	4
	PBT/GF25-DEPAL16-MPP8	51	25	16	8

**Table 2:** Reaction mechanism for all the materials examined in this study. The stoichiometric coefficients are mass-based.

Reactive Blend Components	#	Reaction Equation
PBT	1	PBT → PBT_Melt
	2	PBT_Melt → 0.84 PBT_Res1 + 0.16 PBT_Gas1
	3	PBT_Res1 → 0.12 PBT_Res2 + 0.88 PBT_Gas2
PBT and DEPAL	4	DEPAL → 0.07^a^ DEPAL_Res1 + 0.93^a^ DEPAL_Gas1
	5	PBT_Melt + 3.6 DEPAL → 2.0 PBT_DEPAL_Res1 + 2.6 PBT_DEPAL_Gas1
PBT and MPP	6	MPP → 0.4 MPP_Res1 + 0.6 MPP_Gas1
	7	PBT_Res1 + 0.04 MPP → 0.92 PBT_MPP_Res1 + 0.12 PBT_MPP_Gas1
	8	PBT_MPP_Res1 → 0.07 PBT_MPP_Res2 + 0.93 PBT_MPP_Gas2

^a^ Stoichiometric coefficients obtained from literature [32].

**Table 3:** Kinetics and thermodynamics of melting and decomposition reactions. Positive heats of reaction, h, correspond to endothermic processes.

#	A (s^−1^ or m^3^·kg^−1^·s^−1^)	E (J·mol^−1^)	h (J·kg^−1^)	#	A (s^−1^ or m^3^·kg^−1^·s^−1^)	E (J·mol^−1^)	h (J·kg^−1^)
1	$2.0\times 10^{40}$	$4.00\times 10^{5}$	$6.0\times 10^{4}$	5	$2.0\times 10^{20}$	$3.19\times 10^{5}$	$2.4\times 10^{6}$
2	$2.0\times 10^{25}$	$3.41\times 10^{5}$	$1.4\times 10^{5}$	6	$2.5\times 10^{20}$	$2.88\times 10^{5}$	$6.9\times 10^{5}$
3	$2.4\times 10^{20}$	$2.90\times 10^{5}$	$3.1\times 10^{5}$	7	$2.0\times 10^{20}$	$2.70\times 10^{5}$	$1.5\times 10^{5}$
4	$1.0\times 10^{12}$	$2.09\times 10^{5}$	0	8	$2.5\times 10^{20}$	$2.87\times 10^{5}$	$4.0\times 10^{5}$

**Table 4:** Heat capacities of condensed-phase components.

Component	c (J·kg^−1^·K^−1^)	Component	c (J·kg^−1^·K^−1^)
PBT	−524+5.60×T	DEPAL_Res1	1700
PBT_Melt	2100+0.20×T	PBT_DEPAL_Res1	1700
PBT_Res1	1900+0.10×T	MPP	−990+4.20×T
PBT_Res2	1700	MPP_Res1	1700
GF	(442+1.24×T) ^a^	PBT_MPP_Res1	1900+0.10×T
DEPAL	−2750+11.8×T	PBT_MPP_Res2	1700

^a^ Obtained from the manufacturer [34].

**Table 5:** Heats of combustion of gaseous decomposition products.

Component	h_c_ (J·kg^−1^)	Component	h_c_ (J·kg^−1^)	Component	h_c_ (J·kg^−1^)
PBT_Gas1	$2.1\times 10^{7}$	PBT_DEPAL_Gas1	$2.8\times 10^{7}$	PBT_MPP_Gas1	$3.8\times 10^{7}$
PBT_Gas2	$2.5\times 10^{7}$	MPP_Gas1	$1.8\times 10^{7}$	PBT_MPP_Gas2	$2.5\times 10^{7}$
DEPAL_Gas1	$0.7\times 10^{7}$				
